# Tunable high-order sideband spectra generation using a photonic molecule optomechanical system

**DOI:** 10.1038/srep22920

**Published:** 2016-03-10

**Authors:** Cong Cao, Si-Chen Mi, Yong-Pan Gao, Ling-Yan He, Daquan Yang, Tie-Jun Wang, Ru Zhang, Chuan Wang

**Affiliations:** 1 School of Science, Beijing University of Posts and Telecommunications, Beijing 100876, China; 2 State Key Laboratory of Information Photonics and Optical Communications, Beijing University of Posts and Telecommunications, Beijing 100876, China; 3 School of Information and Communication Engineering, Beijing University of Posts and Telecommunications, Beijing 100876, China; 4 School of Ethnic Minority Education, Beijing University of Posts and Telecommunications, Beijing 100876, China

## Abstract

A tunable high-order sideband spectra generation scheme is presented by using a photonic molecule optomechanical system coupled to a waveguide beyond the perturbation regime. The system is coherently driven by a two-tone laser consisting of a continuous-wave control field and a pulsed driving field which propagates through the waveguide. The frequency spectral feature of the output field is analyzed via numerical simulations, and we confirm that under the condition of intense and nanosecond pulse driving, the output spectrum exhibits the properties of high-order sideband frequency spectra. In the experimentally available parameter range, the output spectrum can be efficiently tuned by the system parameters, including the power of the driving pulse and the coupling rate between the cavities. In addition, analysis of the carrier-envelop phase-dependent effect of high-order sideband generation indicates that the system may present dependence upon the phase of the pulse. This may provide a further insight of the properties of cavity optomechanics in the nonlinear and non-perturbative regime, and may have potential applications in optical frequency comb and communication based on the optomechanical platform.

Cavity optomechanics^1^,^2^ describes the interaction between the electromagnetic radiation and nanomechanical or micromechanical motion, which has been developing rapidly. During the past decades, it leads to various important applications, such as gravitational-wave detection^3^, cooling of mechanical oscillators to the ground-state of motion^4^,^5^,^6^,^7^,^8^,^9^,^10^,^11^, optomechanically induced transparency (OMIT) and slow light^12^,^13^,^14^,^15^,^16^,^17^,^18^, precision measurements^19^,^20^, squeezing of light^21^, quantum information processing^22^,^23^,^24^, and so on.

Most of the recent developments in cavity optomechanics are based on the perturbative interaction between the driving light fields and the optomechanical system. For example, in the context of OMIT, the optomechanical system is coherently driven by both a control field and a probe field. If the strength of the probe field is far less than that of the control field, the perturbation method can be used and the OMIT can be properly described by the linearization of the Heisenberg-Langevin equations. Aside from OMIT, the linearization of optomechanical interaction has also been adopted in many other studies, such as optomechanical dark state^25^ and normal mode splitting^26^.

One key aspect is that if the strength of the probe field becomes comparable with that of the control field, the perturbative description breaks down, and some novel nonlinear and non-perturbative effects come to appear^27^,^28^. Therefore, extending the studies of cavity optomechanics from the linear and perturbation regime to the nonlinear and non-perturbative regime is of great interest. On the other hand, as a natural extension of the generic optomechanical system, the composite optomechanical system which consists of two directly coupled whispering-gallery-mode microcavities (called a photonic molecule^29^,^30^) with optomechanical oscillation in one microcavity has attracted much attention. In the composite system, the interplay between the optomechanical interaction and the tunable photon tunnelling forms the basis of some interesting phenomena, such as phonon lasing^31^,^32^, enhanced quantum nonlinearities^33^, coherent optical wavelength conversion^34^, optomechanical quantum gates^22^,^23^, coherent control of light transmission^35^,^36^,^37^,^38^,^39^,^40^,^41^, chaos^42^, and ground-state cooling of mechanical modes^43^,^44^.

In this work, we investigate the tunable high-order sideband spectra generation using a photonic molecule optomechanical system coupled to a waveguide in the non-perturbation regime. The composite system is coherently driven by a two-tone laser consisting of a continuous-wave (CW) control field and a pulsed driving field which propagates through the waveguide. We analyze the frequency spectral feature of the output field by performing fast Fourier transformation (FFT), and confirm that under the condition of intense and nanosecond pulse driving, the output spectrum exhibits the properties of high-order sideband frequency spectra. We find that the output spectrum can be efficiently tuned by using the power of the driving pulse and the coupling rate between the cavities. In addition, we show the carrier-envelop phase-dependent (CEP-dependent) effect of high-order sideband generation in the output spectra, and the results indicate that the CEP of laser pulse which contains huge numbers of cycles can also cause profound effects. Our study may provide a further insight of the properties of cavity optomechanics in the nonlinear and non-perturbative regime, and may have potential applications in optical frequency comb and communication based on the optomechanical platform^45^. Also the proposed whispering-gallery-mode photonic molecule-waveguide structure is compatible with large-scale integration for implementing complex photonic devices on a chip.

## Results

### Model

**Figure 1:**
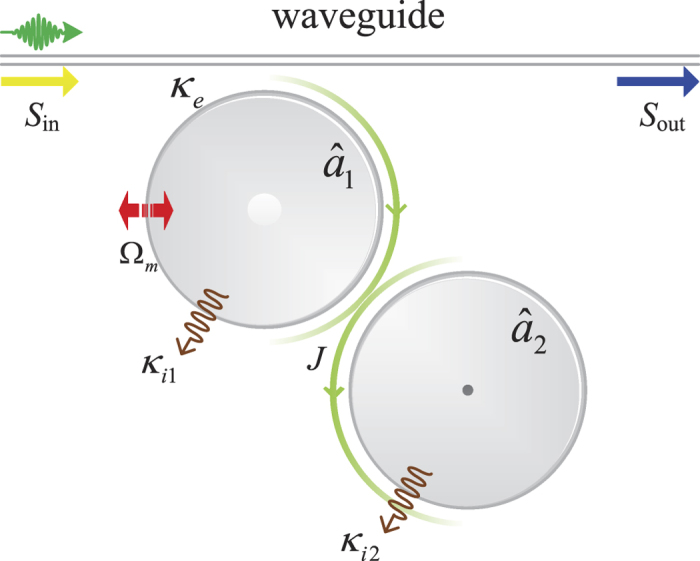
Schematic diagram of a double-cavity photonic molecule optomechanical system coupled to a waveguide. *S*_*in*_ (*S*_*out*_) represents the input (output) field propagating in the waveguide. 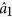 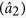 denotes the bosonic annihilation operator of the first (second) cavity field. The first cavity supports a mechanical mode with angular frequency Ω_*m*_. *J* is the cavity-cavity coupling rate, which can be efficiently adjusted by the distance between the cavities. κ_*e*_ denotes the coupling rate between the first cavity and the waveguide. κ_*i*1_ and κ_*i*2_ are the intrinsic cavity decay rates.

As shown in Fig. 1, we consider a system of two directly coupled whispering-gallery-mode microcavities. The first cavity with an effective mass *m* supports an optical mode 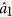 and a mechanical mode with angular frequency Ω_*m*_. The second cavity only supports an optical mode 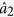 which is coupled to the first cavity through an evanescent field. The cavity-cavity coupling rate *J* can be efficiently tuned by changing the distance between them. A tapered fiber is used to excite the cavity modes as the optical waveguide. The first cavity is side coupled to the fiber with the coupling rate *κ*_*e*_. *S*_*in*_ and *S*_*out*_ represent the input and the output fields propagating in the waveguide, respectively. The Hamiltonian of this composite system can be divided into three parts, i.e.,


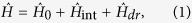


where 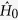, 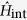 and 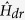 are the free Hamiltonian, interaction Hamiltonian and the driven Hamiltonian, respectively, which can be written as


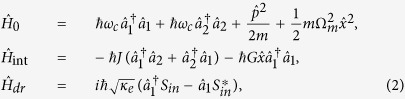


where 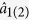 and 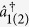 represent the bosonic annihilation and creation operators of the first (second) cavity mode. *ω*_*c*_ is the resonance frequency of the two cavities. 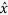 and 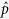 denote the mechanical position and momentum operators. *G* is the optomechanical coupling constant. Here we focus on the mean response of the composite system. Assuming 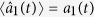, 
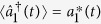
, 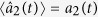, 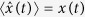, 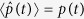, and by using the mean-field approximation 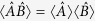, the Heisenberg-Langevin equations of the operators can be reduced to the mean value equations as:


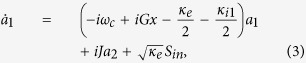



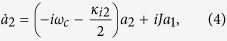



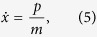



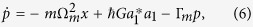


where *κ*_*i*1_ and *κ*_*i*2_ are the cavity intrinsic decay rates. Γ_*m*_ represents the mechanical decay rate, which is introduced classically. The quantum and thermal noise terms are dropped as their mean values are 0. Eqs (3)–(6), 19, 20, 21 are coupled ordinary differential equations of complex functions which describe the time evolution of the composite system.

In the following, we consider the case that the input field contains a CW control field and a pulsed driving field, i.e., 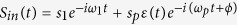. *s*_1_ and *s*_*p*_*ε*(*t*) are the amplitudes of the two fields, which are related to the optical powers propagating in the waveguide by 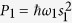 and 
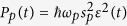
, respectively. *ω*_1_ and *ω*_*p*_ are the frequency of the control field and the mean frequency of the driving pulse, respectively. *ε*(*t*) is the normalized pulse envelope which is assumed to be Gaussian here, i.e., 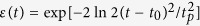, with *t*_0_ being the center time of the pulse and *t*_*p*_ being the full width of half maximum (FWHM) of the intensity envelop. *ϕ* is the so-called CEP of the driving pulse. In this case, the maximum power of the driving pulse is 
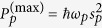
. In a rotating frame at the frequency of *ω*_1_, the evolution equations of the composite system can be rewritten as:


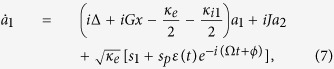



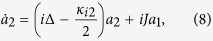



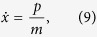



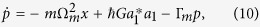


where 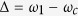 is the detuning between the frequency of the control field *ω*_1_ and the cavity resonance frequency *ω*_*c*_. Ω = *ω*_*p*_ − *ω*_1_ is the detuning between the mean frequency of the driving pulse *ω*_*p*_ and the frequency of the control field *ω*_1_. In such a rotating frame, 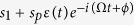 can be seen as the effective input field, and the mean frequency of the effective driving pulse becomes Ω. Here, we set Ω equals to the low-frequency mechanical mode Ω_*m*_, which has been widely employed in OMIT.

### High-order sideband frequency spectra generation

**Figure 2:**
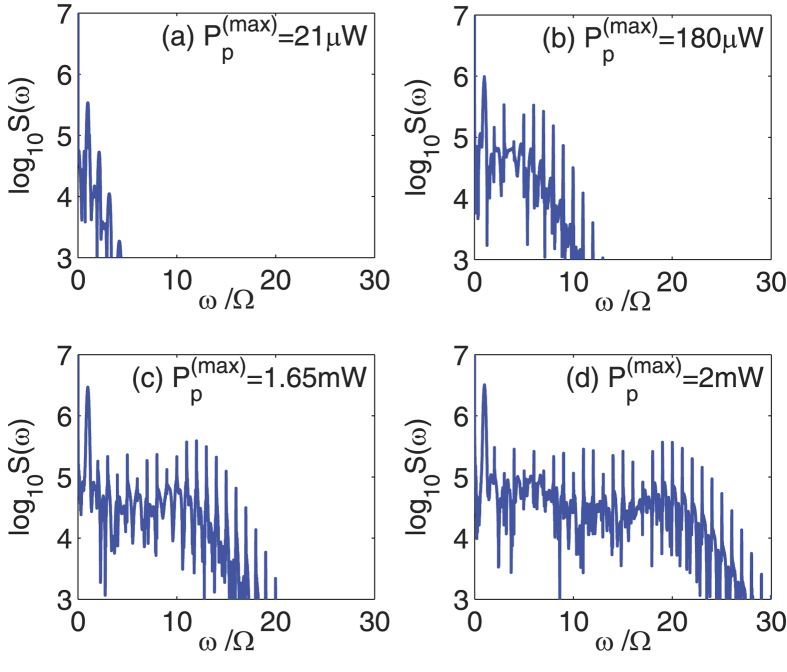
Frequency spectra of the output field with different values of 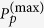 as: (**a**) 
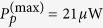
, (**b**) 
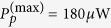
, (**c**) 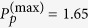 mW, and (**d**) 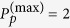 mW. Here the CW control field has wavelength 532 nm and power *P*_1_ = 37.3 *μ*W. The driving pulse has center time *t*_0_ = 0.2 *μ*s, FWHM *t*_*p*_ = 12 ns, and CEP *ϕ* = 0. The cavity-cavity coupling rate is *J* = 2*π* × 0.5 MHz. The other system parameters used for calculations are κ_*e*_ = 2*π* × 45.5 MHz, κ_*i*1_ = κ_*i*2_ = 2*π* × 0.5 MHz, *m* = 2.0 pg, Ω_*m*_ = 1.4 GHz, Γ_*m*_ = 2*π* × 35 kHz, *G* = 485.0 GHz/nm, and Δ = −Ω_*m*_, respectively.

Figure 2 shows the frequency spectra of the output field with different powers of the driving pulse 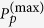 as: (a) 
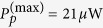
, (b) 
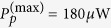
, (c) 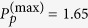 mW, and (d) 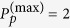 mW. Here the CW control field has wavelength 532 nm and power 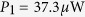, with 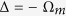. The driving pulse has center time 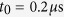, FWHM *t*_*p*_ = 12 ns, and CEP *ϕ* = 0. The cavity-cavity coupling rate is *J* = 2*π* × 0.5 MHz. As the Eqs (7)–(10), 28, 29, 30 describe the evolution of the optical fields in a frame rotating at the frequency *ω*_1_, the output spectra exhibit a frequency shift of *ω*_1_. Moreover, there are positive frequencies and negative frequencies during FFT, and we only show the positive frequency components here. From Fig. 2, one can see that the output spectra contain two input field frequency components (the CW control field *ω*_1_ and the pulsed driving field *ω*_*p*_ = *ω*_1_ + Ω), and a series of new components (higher-order sidebands). That is, when the driving pulse is incident upon the composite system which has been driven by the CW control field, the spectral components with frequencies *ω* = *ω*_1_ ± *n*Ω can be generated in the output field, where *n* = 0,1,2,… represents the order of the sidebands. When the driving pulse is relatively weak, e.g., 
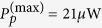
 in Fig. 2(a), there are only a few sidebands appear in the spectrum, and the intensity of individual sideband is decreased rapidly as the order of the sidebands is increased. As the power of the driving pulse is increased, e.g., 
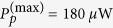
 in Fig. 2(b) and 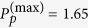 mW in Fig. 2(c), the effect of high-order sideband generation could be observed. When the pulse power is sufficiently high, e.g., 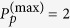 mW in Fig. 2(d), a robust high-order sideband generation can be achieved. In this case, the spectrum decreases rapidly for the first few order sidebands, followed by a plateau where the sideband intensity is slowly varying, and ends up with a sharp cutoff. Such typical spectral feature indicates the non-perturbative nature of high-order sideband generation^27^, which is very similar to the high-order harmonic generation in strongly driven atoms or molecules^46^,^47^.

**Figure 3:**
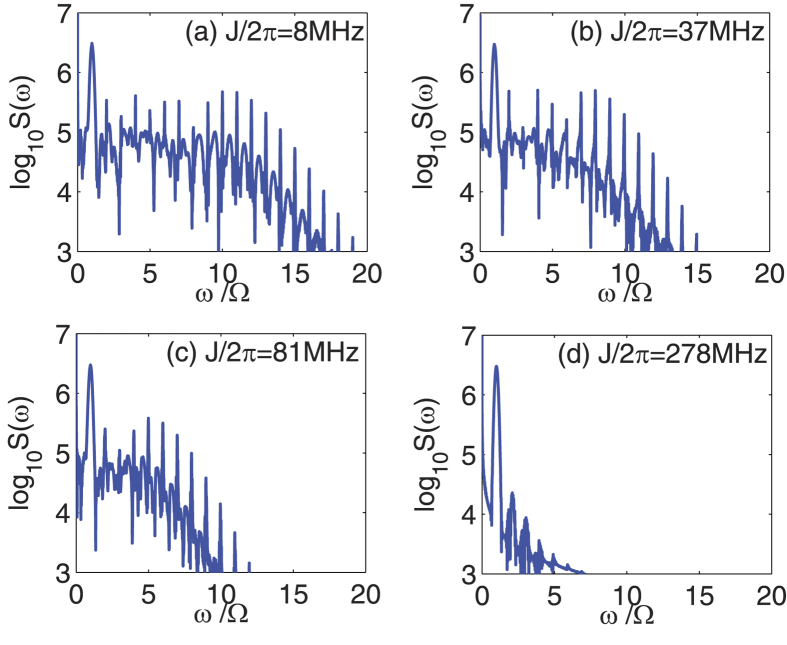
Frequency spectra of the output field with different values of *J* as: (**a**) *J* = 2*π* × 8 MHz, (**b**) *J* = 2*π* × 37 MHz, (**c**) *J* = 2*π* × 81 MHz, and (**d**) *J* = 2*π* × 278 MHz. Here the power of the CW control field is *P*_1_ = 37.3 *μ*W, the maximum power of the driving pulse is 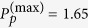 mW, and the other parameters are the same as in Fig. 2.

In experiment, the cavity-cavity coupling rate *J* can be efficiently adjusted by changing the distance between the cavities. In order to show the influence of the parameter *J* on the high-order sideband spectra generation, Fig. 3 shows the frequency spectra of the output field with different values of *J* as: (a) *J* = 2*π* × 8 MHz, (b) *J* = 2*π* × 37 MHz, (c) *J* = 2*π* × 81 MHz, and (d) *J* = 2*π* × 278 MHz. Note that the power of the CW control field is *P*_1_ = 37.3 *μ*W, the maximum power of the driving pulse is 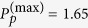 mW, and the other system parameters used for calculations are the same as in Fig. 2. When *J* is relatively small, e.g., *J* = 2*π* × 8 MHz in Fig. 3(a), a lot of high-order sidebands can be obtained and the corresponding sideband intensities are large. With the increment of *J*, e.g., *J* = 2*π* × 37 MHz in Fig. 3(b) and *J* = 2*π* × 81 MHz in Fig. 3(c), the intricate competition between the two kinds of couplings, i.e., the cavity-cavity coupling and the optomechanical coupling, becomes obvious. Due to the linear cavity-cavity coupling indirectly influences the optomechanical coupling strength, the nonlinear effect of high-order sideband generation is decreased^48^,^49^. Last, for a large *J*, e.g., *J* = 2*π* × 278 MHz in Fig. 3(d), the photon tunnelling between the two cavities dominates the evolution process. As a result, only a few sidebands appear in the spectrum and the intensity of individual sideband is decreased rapidly as the sideband order is increased. This phenomenon provides us a potentially useful method to tune the output spectrum by using the photon tunnelling of the coupled cavities.

**Figure 4:**
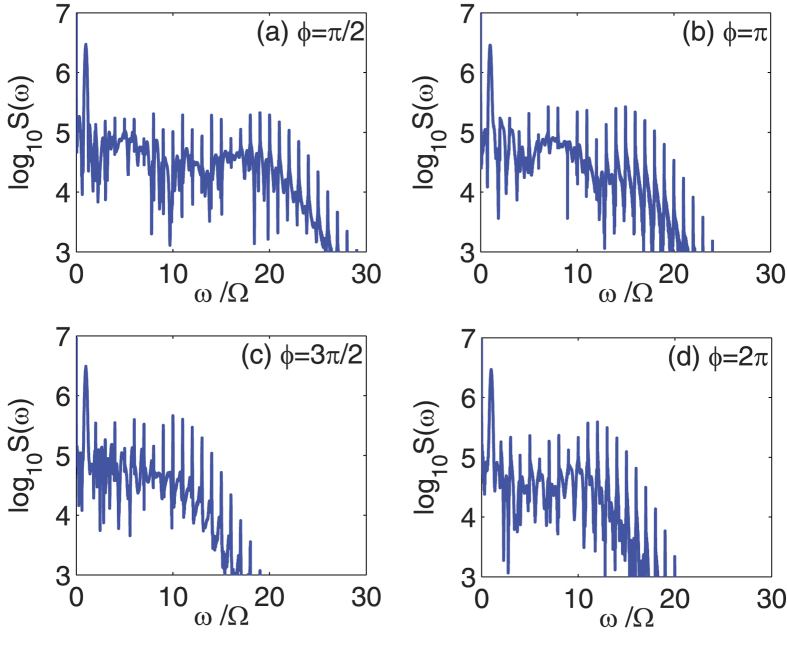
Frequency spectra of the output field with different values of *ϕ* as: (**a**) *ϕ* = *π*/2, (**b**) *ϕ* = *π*, (**c**) *ϕ* = 3*π*/2, and (**d**) *ϕ* = 2*π*. Here we use *P*_1_ = 37.3 *μ*W, 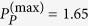 mW, and the other parameters are the same as in Fig. 2.

On the other hand, the CEP is another key parameter in describing the characteristics of the driving pulse. According to Eqs (7)–(10), 28, 29, 30, when the CEP of the driving pulse *ϕ* goes to *ϕ* + 2*π*, the time evolution of the composite system remains unchanged. Thus there is a periodicity upon the CEP, where *ϕ* goes to *ϕ* + 2*π* leads to the same output spectra. However, within a range of 2*π*, the spectra may also be different for different values of *ϕ*. Figure 4 shows such a CEP-dependent effect of high-order sideband generation with different values of *ϕ* as: (a) *ϕ* = *π/*2, (b) *ϕ* = *π*, (c) *ϕ* = 3*π*/2, and (d) *ϕ* = 2*π*. Here we use *P*_1_ = 37.3 *μ*W, 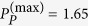 mW, and the other parameters are the same as in Fig. 2. Usually, the CEP only strongly affects the processes involving few-cycle light pulses^50^,^51^. For *t*_*p*_ = 12 ns, the number of cycles in the driving pulse can be estimated to be 
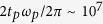
, which means the driving pulse contains huge numbers of cycles and seems almost impossible to have the CEP-dependent effects. However, the dynamics of the composite system in our scheme is in a rotating frame, the mean frequency of the effective driving pulse is Ω, which equals to the low-frequency mechanical mode Ω_*m*_. One can estimate the number of cycles in the effective driving pulse to be 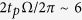 by choosing *t*_*p*_ = 12 ns and Ω = 1.4 GHz, which means the effective driving pulse only contains a few cycles. We can conclude that the CEP of the driving pulse becomes important and can produce obvious influences on the output spectra, even though the driving pulse contains huge numbers of cycles in reality. For longer driving pulses, the influence of the CEP on high-order sideband generation would become smaller^49^.

Before ending, it is worth emphasizing that the linewidth of the high-order sidebands can be well described by the time-frequency uncertainty relation Δ*ω*Δ*t* ~ 2*π*^27^,^28^,^48^,^49^. In our scheme, the driving pulse lasts about 2*t*_*p*_, so Δ*t* ≈ 2*t*_*p*_. Making use of the relationship Δ*ω* ~ 2*π*/Δ*t*, we can roughly estimate the linewidth of the generated high-order sidebands to be Δ*ω* ~ Ω_*m*_/5. Therefore, the high-order sidebands are relatively narrow and clearly resolved in the spectra.

## Summary

In summary, we have theoretically and numerically analyzed the nonlinear optical transmission characteristics in a waveguide-coupled photonic molecule optomechanical system. The composite system is coherently driven by a CW control field and an intense nanosecond driving pulse, and the numerical method is employed instead of the perturbation method in such a non-perturbative regime. By performing FFT, the frequency spectrum of the output field of the system can be obtained. We confirmed that the output spectrum exhibits the properties of high-order sideband frequency spectra, and the typical spectral feature reveals the non-perturbative nature of high-order sideband generation. The results clearly show that the output spectrum is tunable by changing the power of the driving pulse and also by using the photon tunnelling of the coupled cavities. In addition, we investigated the CEP-dependent effect of high-order sideband generation in the output spectra. The results may have potential applications in optical frequency comb and communication based on the optomechanical platform, and may open up a promising perspective for implementing complex photonic devices on a chip.

## Method

In order to show the non-perturbative signals explicitly, we directly solved the evolution equations to study the dynamics of the system. The Runge-Kutta method is employed and the initial conditions are set as: *a*_1_(0) = 0, *a*_2_(0) = 0, *x*(0) = 0, and *p*(0) = 0, which can be achieved by cooling the mechanical mode to the ground-state of motion. The relevant system parameters used for calculations are κ_*e*_ = 2*π* × 45.5 MHz, κ_*i*1_ = κ_*i*2_ = 2*π* × 0.5 MHz, *m* = 2.0 pg, Ω_*m*_ = 1.4 GHz, *G* = 485.0 GHz/nm, Γ_*m*_ = 2*π* × 35 kHz, and Δ = −Ω_*m*_, respectively. All these parameters are within the experimentally available parameter range^52^, and are used through out the paper.

**Figure 5:**
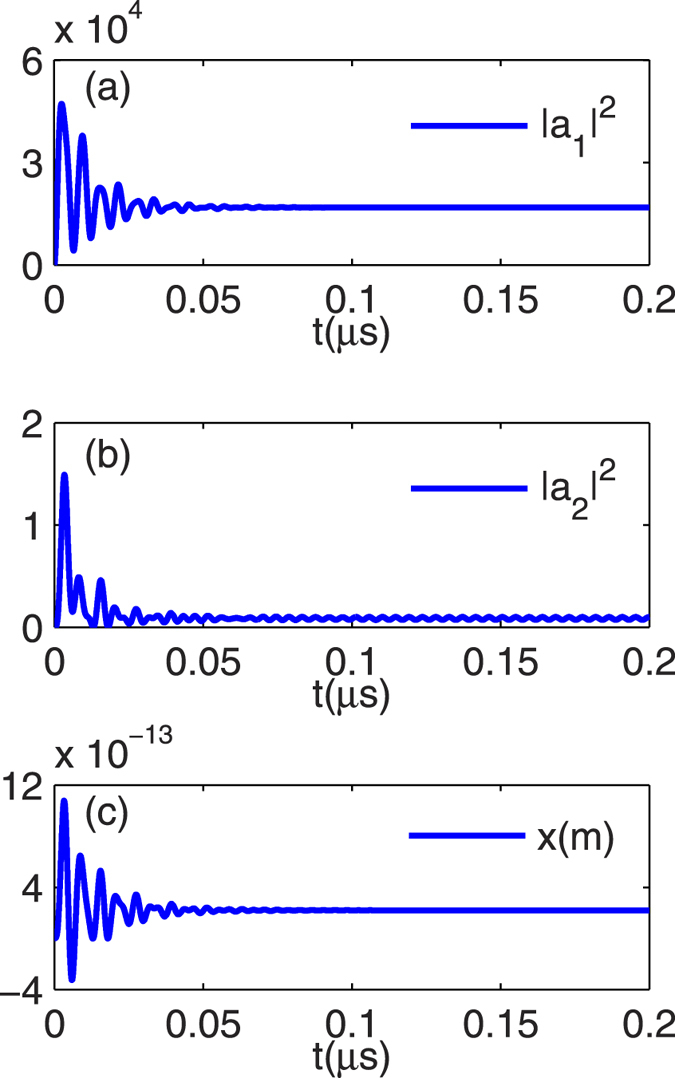
Intensities of the optical fields and the mechanical oscillation in time domain by directly solving Eqs (7)–(10)Eqs (7)–(10)Eqs (7)–(10)Eqs (7)–(10) with *s*_*p*_ = 0 and *J* = 2*π * **× 0.5 MHz.** The time evolution of the (**a**) photon number in the first cavity 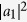, (**b**) photon number in the second cavity 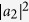, and (**c**) mechanical position *x* in 0.2 *μ*s are shown.

Firstly, we consider *s*_*p*_ = 0 and only a CW control field with wavelength 532 nm and power *P*_1_ = 37.3 *μ*W is incident upon the composite system. In this case, the system would evolve to a steady state provided by the effective field *s*_1_. Figure 5 shows the time evolution of the composite system by solving Eqs (7)–(10), 28, 29, 30 with the numerical method. One can see that after a transient process, the system reaches the steady state at about *t* = 0.1 *μ*s.

Next, the input field contains a CW control field and a nanosecond driving pulse is taken into consideration. The center time of the driving pulse is adjusted to *t*_0_ = 0.2 *μ*s to make sure that when the pulse is incident upon the composite system, the system has reached the steady state. The output field transmitting through the waveguide can be obtained by using the standard input-output formalism as 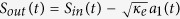. The output spectrum 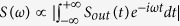 can be numerically obtained by performing FFT on 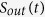, where 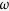 is the spectrometer frequency. Here we do the FFT from *t* = 0.15 *μ*s which is after the composite system reaches the steady state and before the driving pulse is incident upon the composite system, so that the complicated transient process has no influence on the spectral characters.

## Additional Information

**How to cite this article**: Cao, C. *et al.* Tunable high-order sideband spectra generation using a photonic molecule optomechanical system. *Sci. Rep.* **6**, 22920; doi: 10.1038/srep22920 (2016).
